# Driven gyrotropic skyrmion motion through steps in magnetic anisotropy

**DOI:** 10.1038/s41598-019-42929-w

**Published:** 2019-04-25

**Authors:** Yifan Zhou, Rhodri Mansell, Sebastiaan van Dijken

**Affiliations:** NanoSpin, Department of Applied Physics, Aalto University School of Science, P.O. Box 15100, FI-00076 Aalto, Finland

**Keywords:** Magnetic properties and materials, Spintronics, Spintronics, Electronic and spintronic devices

## Abstract

The discovery of magnetic skyrmions in ultrathin heterostructures has led to great interest in possible applications in memory and logic devices. The non-trivial topology of magnetic skyrmions gives rise to a gyrotropic motion, where, under an applied energy gradient a skyrmion gains a component of motion perpendicular to the applied force. So far, device proposals have largely neglected this motion or treated it as a barrier to correct operation. Here, we show that skyrmions can be efficiently moved perpendicular to an energy step created by local changes in the perpendicular magnetic anisotropy. We propose an experimentally-realizable skyrmion racetrack device which uses voltage-controlled magnetic anisotropy to induce a step in magnetic anisotropy and drive a skyrmion unidirectionally using alternating voltage pulses.

## Introduction

Magnetic skyrmions are particle-like spin textures with a non-trivial topological number^1,2^. Several mechanisms can give rise to skyrmion formation in different materials, such as four-spin exchange^3^, dipole-dipole interactions^4^, frustrated exchange interactions^5^ and the Dzyaloshinskii-Moriya interaction (DMI)^6–8^. Amongst these, skyrmions in magnetic heterostructures with interfacial DMI and perpendicular magnetic anisotropy (PMA) have attracted most interest for technological applications^8–12^. The current-induced motion of such skyrmions has been investigated intensively, and the required driving current has been shown to be much lower than that required for domain walls in similar systems^13^. However, the energy required by current-controlled devices is still relatively high per bit compared to approaches based on controlling magnetism through electric fields^14,15^. These devices exploit the ability to electrically control the magnetic anisotropy. For perpendicularly magnetized materials it has been shown that the anisotropy can be controlled by applying voltages across ferromagnet-metal oxide interfaces^16–18^. In most implementations, the electric field at the interface alters the 3d orbital occupation of a magnetic transition metal, modifying the perpendicular magnetic anisotropy^17^, although the largest effects have come from altering the oxidation state at the interface^18^. Voltage-controlled magnetic anisotropy (VCMA) has been combined with skyrmion devices in various proposals to create static and racetrack-like memories^19–23^. However, these proposals, similarly to proposals concerning current-driven skyrmions, do not exploit the gyrotropic motion created by the topology of skyrmions^9^, with few exceptions^24^.

In this work, we demonstrate how skyrmion motion can be induced by a step in magnetic anisotropy, which could be created by VCMA, and propose a device implementation exploiting this effect. We start by studying the motion of skyrmions in an anisotropy gradient^24^, comparing the results of micromagnetic simlations with predictions of the analytical Thiele equation^25^ which describes the motion of a rigid skyrmion. We investigate how different material parameters affect the skyrmion motion. Finally, we demonstrate that a step in anisotropy can provide a similar effect to a gradient and introduce a device that could allow the experimental demonstration of an electric-field controlled skyrmion racetrack memory.

## Results

### Anisotropy gradient-dependent skyrmion motion

**Figure 1:**
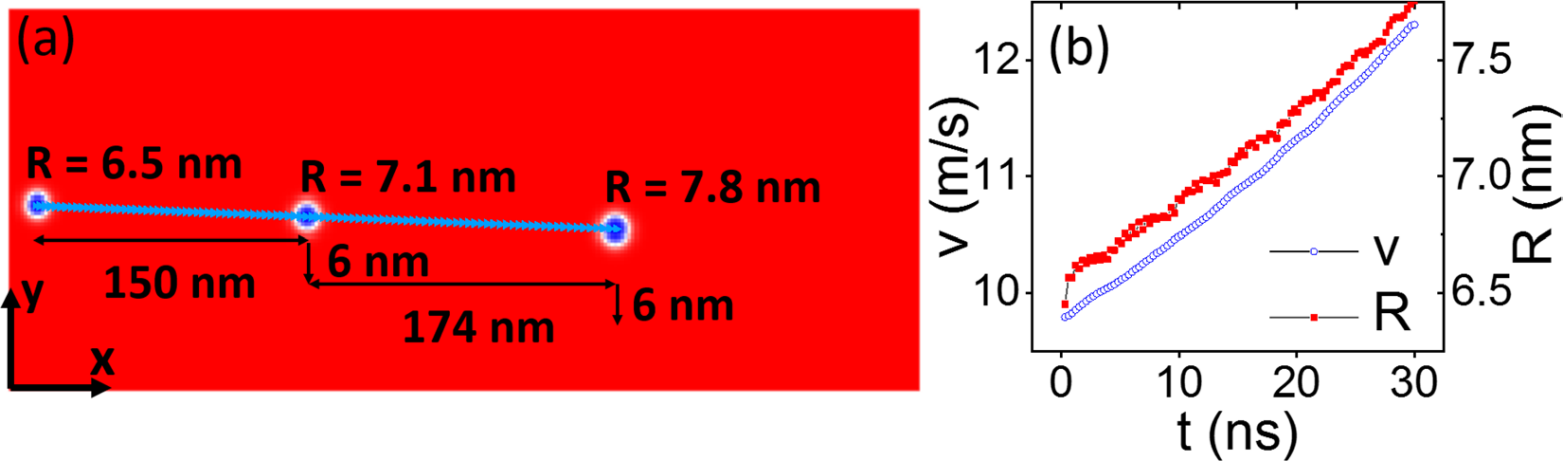
(**a**) Snapshots of skyrmion motion induced by an anisotropy gradient in the *y* direction at 15 ns intervals. The distance travelled and corresponding skyrmion radius are marked at each time interval. The blue line gives the trace of the skyrmion center during 30 ns. (**b**) Skyrmion velocity *v* (blue circles) and radius *R* (red squares) with time *t*.

We first simulate the dynamics of a single skyrmion in a nanotrack with the presence of an anisotropy gradient. Figure 1(a) shows the result of micromagnetic simulations (see methods for details) with a skyrmion shown at three sequential times, 15 ns apart. This simulation uses a rate of change of the uniaxial anisotropy of Δ*K*_*u*_ = 3 × 10^12^ J/m^4^ in the *y* direction, equal to a total change in anisotropy of 6 × 10^5^ J/m^3^ across the nanotrack. The motion of the skyrmion center is plotted onto the nanotrack, showing that it moves nearly perpendicular to the anisotropy gradient. In Fig. 1(b) the skyrmion velocity and radius are shown as a function of time, demonstrating how the skyrmion expands and accelerates as it moves to lower anisotropy. We can compare the simulation with the Thiele equation, which decomposes the motion of a rigid skyrmion under an applied force into gyrotropic and dissipative terms. By assuming that the spin structure of a skyrmion is rigid, we can define ***m***(***r***, *t*) = ***m***(*r* − ***C***(*t*)), where ***r*** is the position vector of the spins to the skyrmion center and ***C***(*t*) is the centre-of-mass coordinate of the structure. The dynamics of a magnetic skyrmion structure can then be described by the Thiele equation^9,25–27^:

1 $${M}_{s}{\gamma }^{-1}{\boldsymbol{G}}\times \dot{{\boldsymbol{C}}}+{M}_{s}{\gamma }^{-1}\alpha {\mathscr{D}}\dot{{\boldsymbol{C}}}={\boldsymbol{F}},$$

where

2 $$\begin{array}{rcl}G & = & \iint {\rm{d}}x{\rm{d}}y{\boldsymbol{m}}\cdot (\frac{\partial {\boldsymbol{m}}}{\partial x}\times \frac{\partial {\boldsymbol{m}}}{\partial y}),\\ {\mathscr{D}} & = & \iint {\rm{d}}x{\rm{d}}y\frac{\partial {\boldsymbol{m}}}{\partial x}\cdot \frac{\partial {\boldsymbol{m}}}{\partial y},\\ {\boldsymbol{F}} & = & -\nabla U.\end{array}$$

Here, ∇*U* is the energy gradient within a skyrmion, and ***F*** is the equivalent driving force. ***G*** = (0, 0, *G*) is the gyromagnetic coupling vector where *G* = 4*π* for a skyrmion. ${\mathscr{D}}$ is called the dissipative force tensor. We model an energy gradient in a VCMA material as a gradient of the uniaxial anisotropy, *K*_*u*_, varying along *y* direction:

3 $$\begin{array}{rcl}{\boldsymbol{F}} & = & -\frac{{\rm{d}}{E}_{{K}_{u}}}{{\rm{d}}{K}_{u}}\frac{{\rm{d}}{K}_{u}}{{\rm{d}}y},\\ {E}_{{K}_{u}} & = & -{K}_{u}\cdot d\iint {\rm{d}}x{\rm{d}}y{({\boldsymbol{m}}\cdot \hat{{\boldsymbol{z}}})}^{2}.\end{array}$$

Here, ${E}_{{K}_{u}}$ is the skyrmion energy due to the uniaxial anisotropy and *d* is the thickness of the magnetic layer and $\hat{{\boldsymbol{z}}}$ is the unit vector in the growth direction. The two-dimensional velocity described in the Thiele equation can then be separated into *x* and *y* axis:

4 $$\begin{array}{l}x:G\cdot {\dot{S}}_{x}-\alpha {\mathscr{D}}{\dot{S}}_{y}=\mathrm{0,}\\ y:G\cdot {\dot{S}}_{y}+\alpha {\mathscr{D}}{\dot{S}}_{x}=F/{M}_{s}{\gamma }^{-1}\mathrm{.}\end{array}$$

where *S*_*x*_ and *S*_*y*_ give the skyrmion position perpendicular and parallel to the anisotropy gradient, respectively, and the dot indicates a time derivative. These equations can be numerically solved as a time-dependent ordinary differential equation. This also leads to the drift angle^22^:

5 $$\tan \,\theta =\frac{|{\dot{S}}_{x}|}{|{\dot{S}}_{y}|}=\frac{G}{\alpha {\mathscr{D}}},$$

where a high value of *θ* indicates motion nearly perpendicular to the gradient. From Eqs (4) and (5) it is clear that a force acting on a skyrmion in one direction will, in general, cause motion both parallel and perpendicular to the force^9^. Further, if the damping term *α* and the dissipative force tensor ${\mathscr{D}}$ are small then the motion will be predominantly perpendicular to the force. Such motion is seen in Fig. 1, where the drift angle is *θ* = 87.9°. We can use the above equations to estimate the drift angle expected from the Thiele equation to check that the theoretical description above accurately describes the simulations. Since the skyrmion radius changes with time, we simplify the estimate by using the average value of the radius during the simulation. We calculate a single value of ${\mathscr{D}}$ for use in the Thiele equation by integrating over the whole system according to Eq. 2 and time averaging over snapshots taken every 0.3 ns. Due to the finite width of the system and the presence of the anisotropy gradient, ${\mathscr{D}}$ is dependent on both the size and position of the skyrmion. Using this method we obtain a drift angle of *θ* = 87.8°, in good agreement with the simulation.

The slight difference can be explained through the fact that in simulations the radius of the skyrmion increases with time, and so changes the dissipative force tensor. Further, the skyrmion expansion also leads to acceleration both in the transverse direction as well as against the anisotropy gradient.

### Influence of material parameters

**Figure 2:**
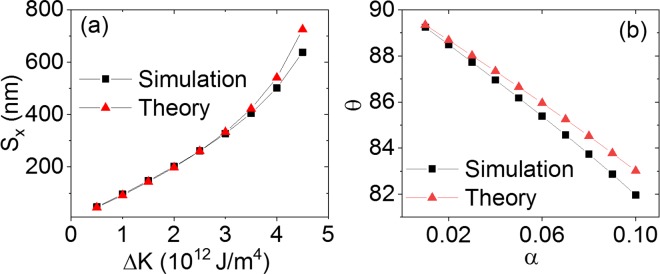
(**a**) Skyrmion travel distance, *S*_*x*_, as a function of the anisotropy gradient, Δ*K*_*u*_. (**b**) Drift angle as a function of the damping constant *α*. Both the theoretical and simulation results for a travel time of 30 ns are shown.

Using the same simulation geometry from Fig. 1, we further investigate how different material parameters influence skyrmion motion under anisotropy gradients. It has be shown using the Thiele equation that an anisotropy energy gradient ${\rm{\Delta }}{E}_{{K}_{u}}$ controls the distance travelled by the skyrmion in *x*, while the damping constant *α* and the dissipative force tensor ${\mathscr{D}}$ determine the drift angle (see Eqs 4 and 5). Figure 2(a) shows the distance of travel, *S*_*x*_, after 30 ns as a function of Δ*K*_*u*_ with a comparison of the simulation and theoretical calculation.

In Fig. 2(b), the drift angle, *θ* is plotted as a function of the damping constant *α*. We find that larger Δ*K*_*u*_ increases the travel distance, and larger *α* decreases the drift angle, which corresponds to the Thiele equation. However, the simulation and theoretical results differ in large Δ*K*_*u*_ and *α*. This is due to the fact that the skyrmion energy is position-dependent in the simulations due to skyrmion expansion, while in the theoretical calculation it is simplified using a fixed skyrmion radius. If Δ*K*_*u*_ > 3 × 10^12^ J/m^4^, the inflation of the skyrmion radius breaks this assumption down^24^. Similarly, for large *α*, the skyrmion radius expands during the simulation. This increases the dissipative force tensor ${\mathscr{D}}$ and leads to a decreased drift angle in the simulations in accordance with Eq. 5. The theoretical drift angles, where ${\mathscr{D}}$ is fixed in each calculation, are consequently larger than in the simulations.

**Figure 3:**
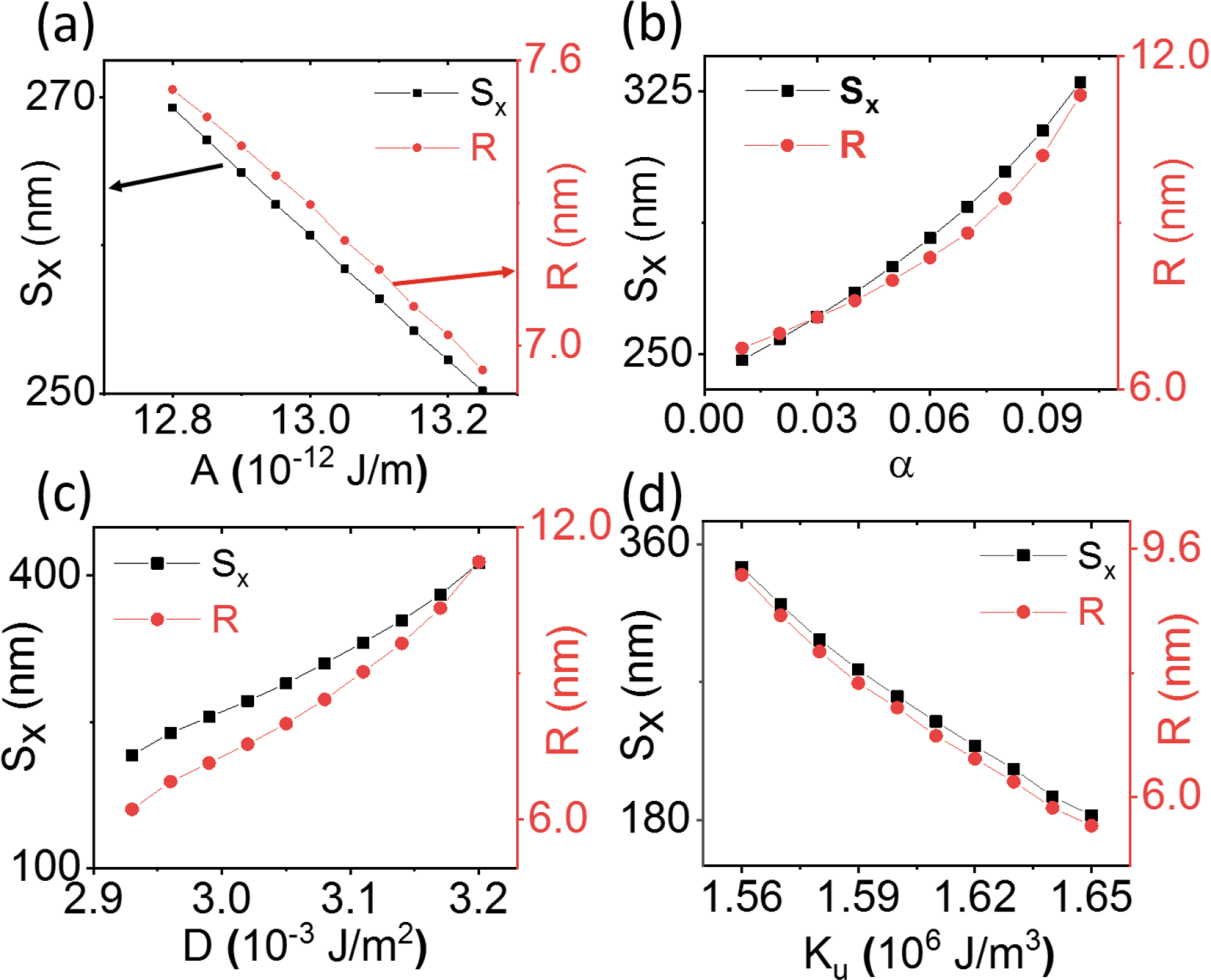
Skyrmion travel distance *S*_*x*_ and skyrmion radius *R* after 30 ns as a function of (**a**) exchange energy *A*, (**b**) damping parameter *α*, (**c**) DMI energy *D*, and (**d**) perpendicular magnetic anisotropy *K*_*u*_. For all plots Δ*K*_*u*_ = 3 × 10^12^ J/m^4^. There is a strong correlation between the skyrmion radius and moving distance.

As discussed above, the skyrmion radius *R* plays an important role in determining the travel distance of a skyrmion after a fixed time. In Fig. 3, we show the travel distance and the final skyrmion radius as a function of (a) exchange energy *A*, (b) damping parameter *α*, (c) DMI energy *D*, and (d) perpendicular magnetic anisotropy *K*_*u*_. The anisotropy gradient Δ*K*_*u*_ is held constant in these simulations. There is a strong correlation between the skyrmion radius at the end of the simulation and the travel distance along *x*.

From the four plots, we can estimate that the relationship between the change in skyrmion radius after 30 ns and each of the parameters roughly follows ${\rm{\Delta }}R\propto \frac{\alpha D}{A{K}_{u}}$. Moreover, larger skyrmions lead to a longer travel distance transverse to the anisotropy gradient, and the distance changes almost linearly with the skyrmion radius. These results show that careful selection of the material parameters will lead to well-defined gyrotropic motion. Moreover, skyrmions have reasonable scaling properties, so that if the device length becomes smaller, the skyrmion size can also be reduced.

### Anisotropy step and device implementation

**Figure 4:**
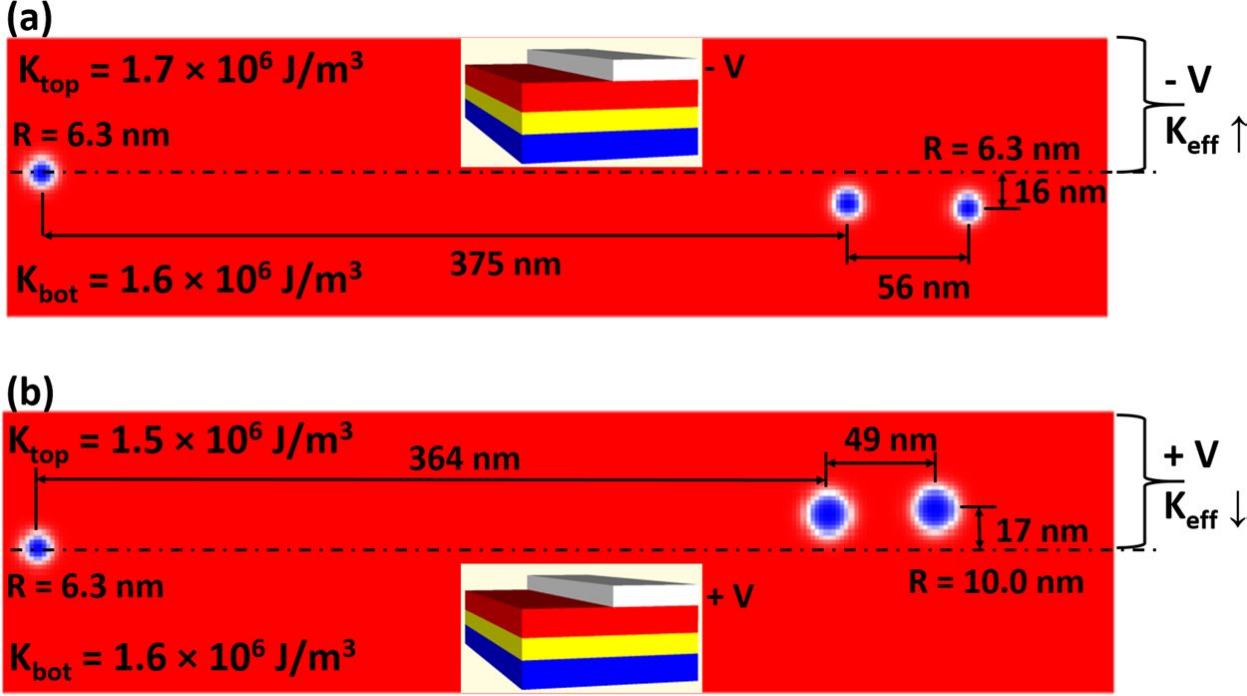
Snapshots of skyrmion motion taken at 50 ns intervals. The horizontal dashed line shows the position of the anisotropy step. The moving distances in *x* and *y* directions, together with the skyrmions radius, are shown in the figures. The sketch of the device is illustrated at the center of each figure, with the fundamental structure from bottom-up: heavy metal layer (blue), ferromagnetic layer (yellow), metal oxide layer (red) and half-width electrode (white). The effective anisotropy is tuned from 1.6 × 10^6^ J/m^3^ to (**a**) 1.7 × 10^6^ J/m^3^ by applying negative voltage and (**b**) 1.5 × 10^6^ J/m^3^ by applying positive voltage.

Having studied the effects of the underlying materials parameters on skyrmion motion in an anisotropy gradient, we now move to a device implementation. For a device, rather than creating a gradient, a step-like profile in anisotropy would be easier to obtain. A schematic of a possible device, where a magnetic nanotrack is half-covered by a top-gate, is shown in the insets of Fig. 4. By applying a voltage on the gate metal (white) across a metal oxide (red) on top of the magnetic layer (yellow) the anisotropy can be varied through VCMA. A skyrmion at the anisotropy step will move along the step (i.e. transverse to the direction of changing anisotropy). Whilst this mechanism can also be described by the Thiele equation, an analytical description of this motion is non-trivial. In our simulations we assume an abrupt anisotropy step. In a real device the step will have some width, at least on the order of the thickness of the oxide layer, but this width is likely to be smaller than the radius of the skyrmion.

There are two approaches to creating skyrmion motion by tuning the anisotropy strength on one side of the nanotrack: increasing the anisotropy by applying a negative voltage, as shown in Fig. 4(a), or decreasing the anisotropy by applying a positive voltage, as shown in Fig. 4(b)^28^. In both cases, the velocity of the skyrmion reduces with time as the skyrmion moves at a small angle away from the anisotropy step. Further, the skyrmions in both anisotropy configurations travel similar distances with comparable drift angles. However, there are also differences. For the anisotropy step in Fig. 4(a), the skyrmion maintains its size during motion, as the skyrmion remains in the initial low anisotropy region. For the step in Fig. 4(b), the skyrmion enlarges continuously while moving into the area with a lower anisotropy value. This difference in behavior suggests that a controlled increase of magnetic anisotropy would create more consistent skyrmion motion by maintaining the skyrmion size.

**Figure 5:**
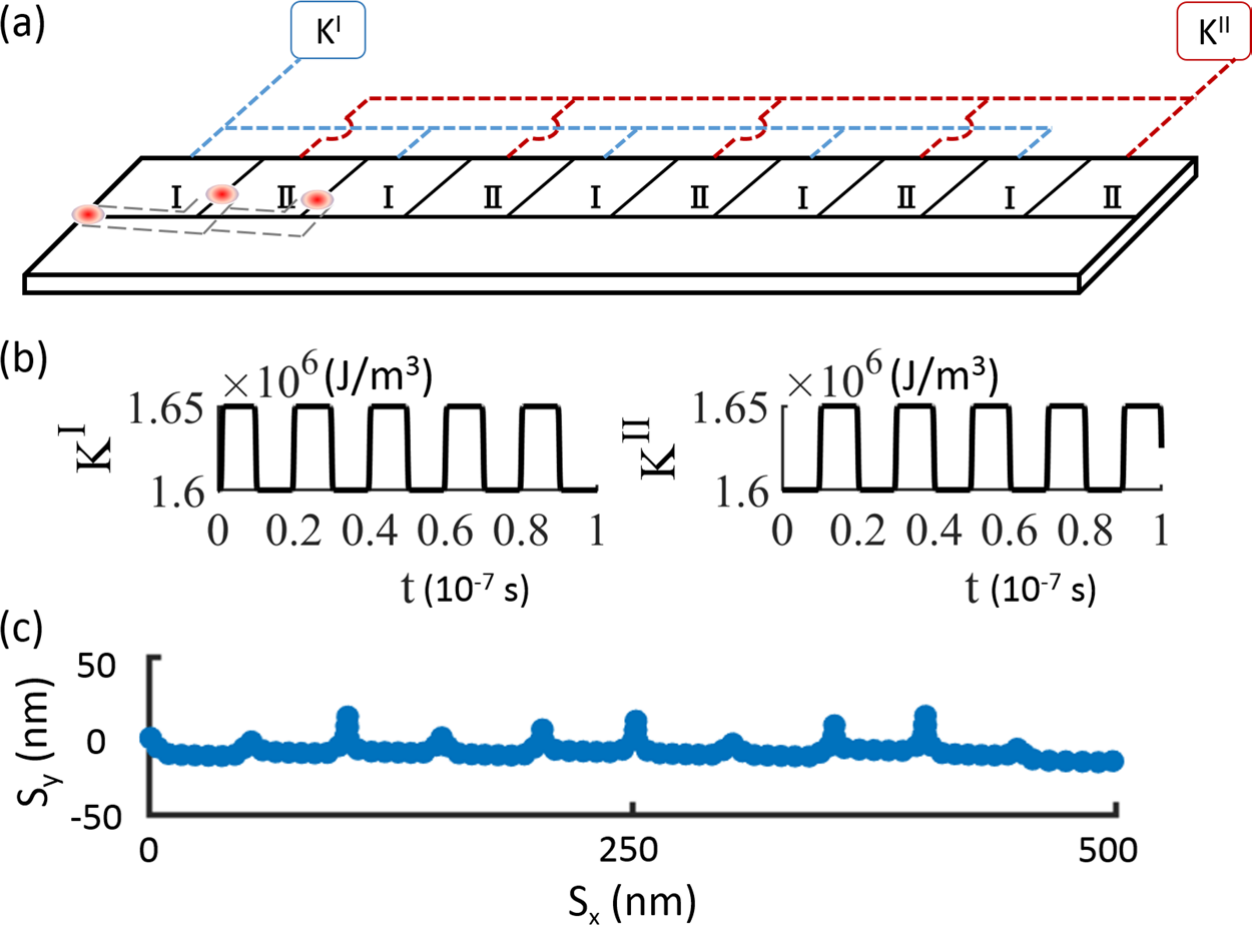
(**a**) Proposed device for moving skyrmions by voltage pulses. Two groups of electrodes are linked in parallel, labeled I and II, with the electrical connections shown by blue and red dashed lines. The voltage pulses are applied alternately on the two groups of electrodes. The dashed line attaching the skyrmions illustrates their motion during the first two pulses. (**b**) The time sequence of anisotropy pulses in the simulation changing the anisotropy in the two blocks by 5 × 10^4^ J/m^3^. (**c**) The motion of a skyrmion during 10 voltage pulses, as extracted from the simulation.

Based on the anisotropy step simulations, a device implementation for a skyrmion racetrack through VCMA is shown in Fig. 5(a). The magnetic nanotrack is covered by an array of ten gate electrodes on one side of the track. The size of each electrode is 50 nm × 50 nm, forming a total length of 500 nm. Negative voltage pulses with a duration of 10 ns are applied sequentially on two sets of alternating and parallel-connected electrode blocks (1 and 2). The simulation is initialized with a skyrmion placed at the left edge of the first electrode as shown in Fig. 5(a). By applying a negative voltage on electrode block 1, regions with higher magnetic anisotropy are periodically created along the nanotrack. This causes the skyrmion to move along the voltage-induced anisotropy step to the first boundary between regions 1 and 2. Then, the voltage on electrode block 1 is turned off, and the voltage on electrodes 2 is turned on. This reverses the modulation of magnetic anisotropy, as shown in Fig. 5(b), and forces the skyrmion to move along the newly formed anisotropy step to the next boundary between electrodes 2 and 1. Because the voltage pulse is longer than the time it takes for the skyrmion to travel 50 nm, the skyrmion moves around the corner of the anisotropy step, at the end of each pulse. When the voltage is switched between the electrode blocks, the skyrmion moves back to the middle and continues its travel along the *x* axis. By alternating the voltage pulses on the two electrode blocks, the skyrmion moves forward one block per pulse, thus giving rise to a synchronous unidirectional movement along the nanotrack, as shown in Fig. 5(c). With these parameters, the skyrmion traverses the 500 nm long device in 100 ns, and the skyrmion is always at least 20 nm from the edge of the nanotrack to avoid any edge effects. A supplementary video showing the device operation is available online.

## Discussion

In the above simulations we have used an anisotropy step of 5 × 10^4^ J/m^3^, corresponding to a change in interfacial anisotropy of 2 × 10^−5^ J/m^2^ at the ferromagnet/metal oxide interface. Assuming a moderate electric field across the interface of 200 MV/m, easily achievable in real devices, this leads to a minimum required VCMA effect of 100 fJ/Vm, a magnitude which has been demonstrated in a variety of systems^18,29^. Whilst we do not specify the skyrmion creation or readout here, all electrical creation and detection methods have already been proposed elsewhere^29,30^.

A key materials feature for the implementation of such a device is low damping. The deviation away from the anisotropy step on each voltage pulse is largely controlled by this parameter. In this paper we have used a value of 0.03 for the device simulations, which is relatively low for perpendicularly magnetized heterostructures, but is similar to that found in CoFeB based systems^31,32^.

We have shown here that a skyrmion can efficiently move transverse to an anisotropy step due to the topology-induced gyrotropic motion. The underlying material parameters affect the transverse motion, in, particular by determining the skyrmion radius, which is closely linked to the velocity of the skyrmion. The gyrotropic motion can be exploited to create a technologically feasible device, where a skyrmion is moves synchronously along a series of magnetic anisotropy steps in a fully electrically controlled skyrmion racetrack. Implementation of the proposed device could lead to a new class of low energy data storage devices.

## Methods

### Analytical expressions

The Hamiltonian of the magnetic skyrmion system includes Heisenberg exchange, the Dzyaloshinskii-Moriya interaction, the Zeeman interaction and an uniaxial anisotropy term, given by:

6 $$ {\mathcal H} =-\sum _{i\ne j}[{J}_{ij}{{\boldsymbol{m}}}_{i}\cdot {{\boldsymbol{m}}}_{j}+{{\boldsymbol{D}}}_{ij}\cdot ({{\boldsymbol{m}}}_{i}\times {{\boldsymbol{m}}}_{j})]-\sum _{i}{\mu }_{s}{{\boldsymbol{m}}}_{i}\cdot {{\boldsymbol{B}}}_{{\rm{app}}}-{k}_{u}\sum _{i}{({{\boldsymbol{m}}}_{i}\cdot {\boldsymbol{e}})}^{2},$$

where ***m***_*i*_ is the three dimensional unit spin vector, defined as ***M***_*s*_/|***M***_*s*_|. ***J***_*ij*_ is the exchange constant, ***D***_*ij*_ the DMI vector, *μ*_*s*_ the local atomic magnetic moment, ***B***_*app*_ the applied magnetic field, *k*_*u*_ the uniaxial anisotropy constant and *e* the direction of easy axis.

The time evolution of a magnetic moment at zero temperature is described by the Laudau-Lifshitz-Gilbert (LLG) equation, written as:

7 $$\frac{{\rm{d}}{\boldsymbol{m}}}{{\rm{d}}t}=-\,\gamma {\boldsymbol{m}}\times {{\boldsymbol{B}}}_{{\rm{eff}}}+\alpha ({\boldsymbol{m}}\times \frac{{\rm{d}}{\boldsymbol{m}}}{{\rm{d}}t}),$$

where *γ* is the gyromagnetic ratio, *α* is the damping constant and ${{\boldsymbol{B}}}_{{\rm{eff}}}=-\,\frac{\partial  {\mathcal H} }{\partial {\boldsymbol{m}}}$ is the effective magnetic field.

### Micromagnetic simulations

The numerical simulations are performed by the open-source software package MuMax3^33^. The nanotrack in all simulations is 512 nm × 200 nm × 0.4 nm using a 2 nm × 2 nm × 0.4 nm mesh. We use the following material parameters, unless explicitly varied as discussed in the main text: saturation magnetization *M*_*s*_ = 1.2 × 10^6^ A/m, exchange constant *A* = 13 × 10^−12^ J/m, damping parameter *α* = 0.03, DMI constant *D* = 3 × 10^−3^ J/m^2^, central uniaxial anisotropy *K*_*u*_ = 1.6 × 10^6^ J/m^3^, where we use the interfacial DMI defined within the MuMax3 package^33^.

## Data Availability

The scripts and datasets generated during the current study are available from the corresponding author on reasonable request.
